# Associations of Urinary Perchlorate, Nitrate, and Thiocyanate with Female Infertility and Mediation of Obesity: Insights from NHANES 2013–2018

**DOI:** 10.3390/toxics13010015

**Published:** 2024-12-26

**Authors:** Lan Zhang, Yuhe Peng, Yue Song, Yu Zhang, Qi Qin, Mengya Ying, Yiyun Bi, Ping Yin

**Affiliations:** Department of Epidemiology and Biostatistics, School of Public Health, Tongji Medical College, Huazhong University of Science and Technology, Wuhan 430030, China; calva_lan@163.com (L.Z.); p2yu303@163.com (Y.P.); m202275511@hust.edu.cn (Y.S.); zhangyu81717@163.com (Y.Z.); m202275593@hust.edu.cn (Q.Q.); ymy199661@163.com (M.Y.); byy112ybb@163.com (Y.B.)

**Keywords:** perchlorate, nitrate, thiocyanate, female infertility, obesity, mediation

## Abstract

Classified as endocrine disrupting chemicals (EDCs), perchlorate, nitrate, and thiocyanate have been implicated with obesity and reproductive disorders. This study used three cycles of the National Health and Nutrition Examination Survey (NHANES 2013–2018); 813 women of reproductive age were finally included. We used multivariable logistic regression to analyze the associations between the three anions and obesity and infertility. Subsequently, we performed mediation analysis to explore the potential mediating effect of obesity on infertility in association with anion exposure. Increased concentrations of perchlorate and nitrate showed inverse correlations with the risk of obesity (OR = 0.73, 95% CI: 0.55–0.96; OR = 0.59, 95% CI: 0.40–0.87). Perchlorate was negatively associated with infertility (OR = 0.68, 95% CI: 0.51–0.91), and obesity was a mediator in association between perchlorate and infertility. These findings suggest that women of reproductive age may be protected from obesity and infertility by exposure to perchlorate and nitrate, with obesity acting as a moderating factor in the observed association. This study provides a valuable understanding of the complex links between environmental contaminants, obesity, and reproductive health, and identifies potential strategies to reduce the risk of infertility and improve women’s health.

## 1. Introduction

Female infertility, defined as the inability to achieve pregnancy after 12 months or more of regular, unprotected sexual intercourse [1], influences 5.8% to 8.1% women in the US, and is increasing year over year [2,3]. This condition not only profoundly impacts women’s reproductive and cardiovascular health, but also exerts significant social and psychological effects on individuals, families, and society, making it a critical public health issue [4,5].

Obesity is defined by the World Health Organization (WHO) as having a body mass index (BMI) greater than 30 kg/m^2^, a condition that affects approximately 800 million people worldwide [6,7]. Obesity rates continue to rise globally every year [8]. About 42% adults in the US are identified as having obesity [9], with the prevalence among women of reproductive age reaching 23% [10]. Obesity in women can lead to a number of reproductive problems, including menstrual irregularities, endometrial abnormalities, and infertility. Additionally, women with obesity are more susceptible to pregnancy complications, including hypertension and gestational diabetes [11,12].

In the United States, perchlorate, thiocyanate, and nitrate in food and the environment are regulated by the Environmental Protection Agency (EPA) and the Food and Drug Administration (FDA). Numerous studies have confirmed that perchlorate, nitrate, and thiocyanate are common environmental endocrine disruptors with significant health impacts [13,14,15]. Perchlorate is widely used in military and industrial sectors, including aerospace, fireworks, and explosives, with environmental exposure mainly through contaminated food and drinking water [16]. Nitrate is widely used in agriculture and food preservation, with dietary intake, particularly from contaminated leafy vegetables and processed foods, being the primary source of exposure [17,18]. Thiocyanate, a cyanide metabolite, is mainly found in cruciferous vegetables (such as broccoli and kale), leafy greens, and dairy products, with tobacco exposure also being a major source [19,20]. Studies have emphasized that the levels of perchlorate, nitrate and thiocyanate in urine can serve as indicators of previous bodily exposure [21]. These three anions are known to disrupt thyroid function by competitively inhibiting iodine uptake at the sodium iodide symporter, thereby exerting endocrine-disrupting effects [22,23]. Research indicated that endocrine disruption caused by thyroid hormone dysfunction affects the hypothalamic–pituitary–ovarian (HPO) axis, thereby influencing female reproduction [24]. Previous biomonitoring reports showed higher exposure to perchlorate, nitrate, and thiocyanate in women. These reports underscore the need for further investigation into the specific risks posed by these chemicals, particularly in relation to reproductive health and endocrine function [25].

Previous studies have shown negative associations between urinary nitrate and obesity, while thiocyanate showed the opposite effect [26]. Obesity can disrupt the HPO axis through mechanisms such as insulin resistance and increased androgen production, potentially leading to infertility and negatively affecting women’s reproductive capacity [27]. Based on these findings, we hypothesize that obesity may mediate the relationship between perchlorate, nitrate, thiocyanate, and infertility. Previous studies have confirmed the association between these anions and the risk of metabolic disorders [28,29,30,31,32]; some studies found that perchlorate was associated with increasing birthweight, while exposure to the baby food and breastfed infants were not associated with adverse outcomes [33,34,35]. These studies suggest that perchlorate may have effects on reproductive health, but the specific association remains unclear. Additionally, the extent to which obesity mediates this association remains largely unexplored, requiring further investigation into the associations between EDCs and obesity and infertility.

This cross-sectional study used data from the National Health and Nutrition Examination Survey (NHANES). We explored the association between perchlorate, nitrate, and thiocyanate and obesity and infertility among women of reproductive age in the United States. Additionally, we performed stratified analyses based on age, marital status, alcohol consumption, and obesity to further investigate the relationship between exposure to these endocrine disruptors and infertility. Finally, we performed mediation analysis to assess the indicator effect of obesity in these associations, providing valuable epidemiological insights for future mechanistic research.

## 2. Materials and Methods

### 2.1. Study Design

This cross-sectional study used data combined from three cycles of NHANES: 2013–2014, 2015–2016, and 2017–2018. Conducted by the National Center for Health Statistics (NCHS) of the Centers for Disease Control and Prevention, NHANES employed a complex multistage probability sampling design to select a representative sample of the U.S. civilian, non-institutionalized population for assessing the health and nutritional status of American children and adults. Participants provided multiple biological samples during physical examinations, including blood and urine, which were used to analyze the associations between environmental exposures and health outcomes. The NHANES protocol was reviewed and approved by the NCHS Research Ethics Review Board, all participants signed informed written consent.

**Figure 1:**
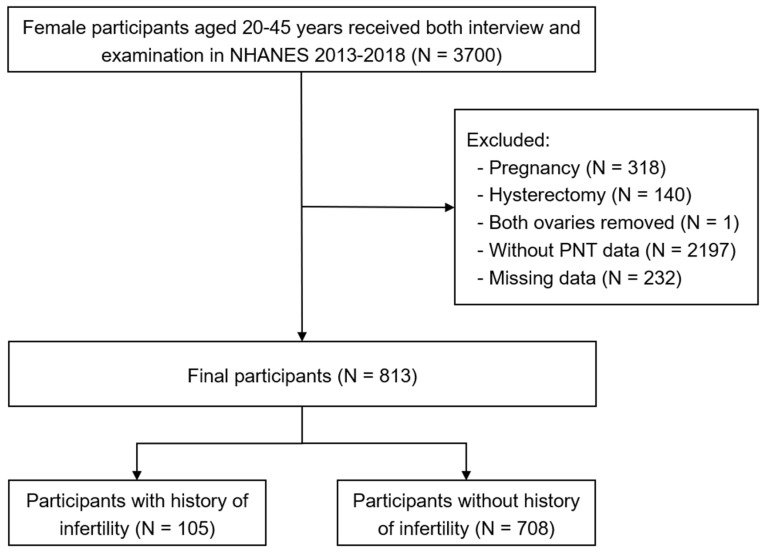
Flowchart for this study. NHANES, National Health and Nutrition Examination Survey; PNT, perchlorate, nitrate, and thiocyanate.

A total of 29,400 participants were included. Participants were excluded based on the following criteria: (1) males (N = 14,452); (2) age under 20 or over 45 years (N = 11,093); (3) participants who completed only the interview portion (N = 155); (4) pregnant or breastfeeding women (N = 318); (5) history of hysterectomy (N = 140); (6) bilateral oophorectomy (N = 1); (7) missing urinary perchlorate, nitrate, or thiocyanate measurements (N = 2197); and (8) incomplete covariate or outcome data (N = 232). Finally, 813 participants were included. Details are presented in Figure 1.

### 2.2. Exposure Ascertainment

Trained survey personnel collected 250 mL random urine samples from participants following standardized procedures outlined in the NHANES laboratory procedures manual. The urine samples were initially processed and preserved under suitable conditions (−20 °C or −30 °C). Perchlorate, nitrate, and thiocyanate concentrations in the urine samples were quantified using ion chromatography and electrospray tandem mass spectrometry. Chromatographic separation was conducted with an IonPac AS16 column using sodium hydroxide as the eluent. The detection limits (LOD) were 0.05 ng/mL for urinary perchlorate, 700 ng/mL for urinary nitrate, and 20 ng/mL for urinary thiocyanate. Analyte concentrations below the LOD were assigned a value of the square root of LOD/2. Further methodological details are provided in the NHANES laboratory methods manual.

### 2.3. Outcome Ascertainment

Infertility was assessed using responses from two questions in reproductive health questionnaire: (1) “Have you ever tried to get pregnant for at least a year without becoming pregnant?” and (2) “Have you ever seen a doctor or other healthcare provider because you were unable to get pregnant?” Individuals who answered “yes” to either question were classified as having infertility, while those who answered “no” were assigned to the control group. To ensure the accuracy and completeness of the data, participants who refused to answer or were unsure were excluded from the analysis.

### 2.4. Mediator Ascertainment

Obesity may serve as a mediator in the association between perchlorate, nitrate, thiocyanate, and infertility, and was therefore treated as a mediator variable in this study. BMI data were collected through physical examinations, and calculated by dividing weight (kg) by height squared (m^2^). According to WHO standards, participants with a BMI of ≥30 kg/m^2^ were classified as obese.

### 2.5. Covariates

The covariates included in the analysis included age (continuous variable), race/ethnicity (non-Hispanic White, non-Hispanic Black, and Others), education level (less than high school, high school or equivalent, and above high school), economic status (low, middle, and high income), alcohol consumption (yes and no), smoking status (yes and no), marital status (living alone and cohabitating), physical activity (sedentary, insufficient, moderate, and high), diabetes (yes and no), hypertension (yes and no), regularity of menstrual cycles (yes and no), use of contraceptive pills (yes and no), and use of female hormone medication (yes and no).

Covariate information was gathered through interviews or questionnaires. Economic status was categorized by the poverty–income ratio (PIR), PIR < 1.3 was considered poverty, PIR 1.3–3.5 was considered normal, and PIR > 3.5 was considered wealthy. Consuming more than 12 alcoholic drinks in the past year was considered alcohol consumption. Smoking was characterized as having smoked more than 100 cigarettes. Marital status was classified based on whether the participant lived with a partner; those who were married or cohabitating were classified as cohabitating [36]. According to weekly leisure-time metabolic equivalent (MET) minutes, physical activity was divided into four groups by 500 and 1000 min [37].

### 2.6. Statistical Analysis

General characteristics were presented as mean (standard deviation, SD), median (interquartile range, IQR) or number (percentage) where appropriate. Continuous variables between groups were compared using the Student’s *t*-test and the Mann–Whitney U test. Categorical variables were compared between the infertility and non-infertility groups using Chi-square or Fisher’s exact tests. To adjust for differences in urine dilution, perchlorate, nitrate, and thiocyanate were adjusted for urinary creatinine prior to analysis (urinary perchlorate: µg/g creatinine; urinary nitrate and thiocyanate: mg/g creatinine) [23]. Creatinine-adjusted urinary perchlorate, nitrate, and thiocyanate were log-transformed to normalize their distribution. We used Spearman correlation analysis to assess the correlation between the three anions. The associations between three anions and outcomes, as well as the odds ratios (OR) and corresponding 95% confidence intervals (95% CI) were examined using multivariable logistic regression models. Several models were established: In Model 1, we adjusted for age and race, we further adjusted demographic factors such as education level and economic status in Model 2, and Model 3 additionally adjusted for diabetes, hypertension, menstrual cycle regularity, contraceptive use, and use of female hormone medications. The analysis accounted for the complex, multistage probability design of NHANES by incorporating appropriate sample weights, in accordance with NHANES analytic guidelines. Stratified analyses were also performed based on age (20–35, 36–45), marital status (living alone, cohabitating), alcohol consumption (yes and no), and obesity (yes and no). Finally, we used the R package “lavaan” to perform mediation analysis.

Additionally, we performed some sensitivity analyses to validate our findings. First, we conducted multivariable logistic regression without applying NHANES weights (sensitivity analysis i). Second, based on Model 3, we further adjusted for perchlorate, nitrate, and thiocyanate simultaneously, in addition to the covariates already included in Model 3 (sensitivity analysis ii). Third, instead of adjusting analyte concentrations for creatinine, we adjusted it as a covariate in Model 3 (sensitivity analysis iii). Fourth, participants with missing covariate data were not excluded; instead, multiple imputation was used to account for missing values (sensitivity analysis iv). Finally, we repeated the analysis after excluding extreme values from the top and bottom 1% (sensitivity analysis v).

All data analyses were used R version 4.3.3, and a bilateral *p*-value of <0.05 was regarded as indicative of statistical significance.

## 3. Results

### 3.1. Characteristics of Participants

Table 1 shows the baseline characteristics of the participants. A total of 813 reproductive-aged women from the United States were included in this study, with 105 participants (12.9%) diagnosed with infertility. Participants in the infertility group were older (*p* = 0.001), with a higher proportion living alone (74.3%). The prevalence of obesity (60.0%), diabetes (7.6%), and hypertension (24.8%) was similarly higher in infertility group. The three anions were detected above the detection limit in more than 99% of participants. After adjusted by urine creatine, perchlorate levels showed a statistically significant difference between the infertility and control groups (*p* = 0.008). The three anions were significantly correlated (*p* < 0.001); details are shown in Figure S1.

### 3.2. Associations Between Perchlorate, Nitrate, and Thiocyanate Exposures and Obesity

The association between the three anions and obesity are summarized in Table 2. In Model 1, both urinary perchlorate (OR = 0.70; 95% CI: 0.53, 0.93; *p* = 0.017) and urinary nitrate (OR = 0.60; 95% CI: 0.40, 0.90; *p* = 0.018) were negatively associated with obesity for each unit increase in ln-transformed concentrations. Participants in the highest quartile of perchlorate and nitrate had a lower risk of obesity than the lowest quartile (perchlorate OR = 0.50; 95% CI: 0.27, 0.91; *p* = 0.028; nitrate OR = 0.45; 95% CI: 0.24, 0.84; *p* = 0.018). After adjusting for covariates in Models 2 and 3, the associations of perchlorate and nitrate with a reduced risk of obesity remained statistically significant. However, we did not find any significant association between thiocyanate and obesity in this study.

### 3.3. Associations Between Perchlorate, Nitrate, and Thiocyanate Exposures and Infertility

Table 3 presents the association between the three anions and infertility. In Model 1, each unit increase in urinary perchlorate (ln-transformed) was negatively associated with infertility (OR = 0.66; 95% CI: 0.50, 0.88; *p* = 0.007). Participants in the highest quartile of perchlorate had a significantly lower risk of infertility than the lowest quartile (OR = 0.46; 95% CI: 0.23, 0.94; *p* = 0.040). After adjusting for demographic factors, perchlorate remained negatively associated with infertility, both for each unit increase and for the highest quartile concentration. In the Model 3, each unit increase in log-transformed urinary perchlorate remained significantly associated with infertility (OR = 0.68; 95% CI: 0.51, 0.91; *p* = 0.016), and the highest quartile had a lower risk of infertility (OR = 0.45; 95% CI: 0.23, 0.90; *p* = 0.034), with a linear trend observed (*p* = 0.013). Results were largely consistent with the main analysis in sensitivity analyses (Table S1).

**Figure 2:**
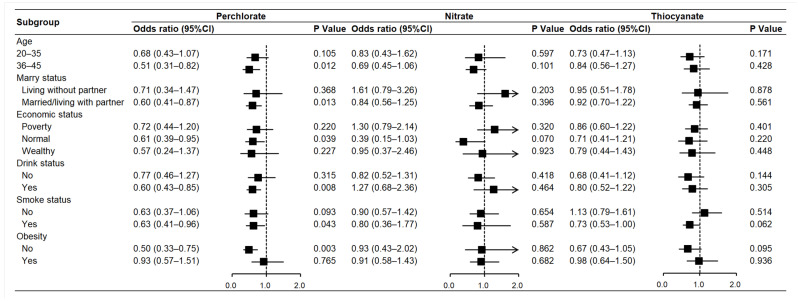
Subgroup analysis of three anions and infertility. Models were adjusted for age (continuous variable), education level, marital status, economic status, alcohol consumption, smoke status, physical activity, diabetes, hypertension, menstrual cycle regularity, contraceptive pills, and female hormones.

Figure 2 shows the results of subgroup analysis. Higher levels of perchlorate were negatively associated with infertility in most subgroups, consistent with the primary findings. Specifically, each unit increase in perchlorate was associated with a lower risk of infertility in the 36–45 age group (OR = 0.51; 95% CI: 0.31, 0.82; *p* = 0.012), the cohabitating group (OR = 0.60; 95% CI: 0.41, 0.87; *p* = 0.013), the middle-income group (OR = 0.61; 95% CI: 0.39, 0.95; *p* = 0.039), the alcohol consumption group (OR = 0.60; 95% CI: 0.43, 0.85; *p* = 0.008), the smoking group (OR = 0.63; 95% CI: 0.41, 0.96; *p* = 0.043), and the non-obese group (OR = 0.50; 95% CI: 0.33, 0.75; *p* = 0.003).

### 3.4. Mediation Analysis for Association of Perchlorate with Infertility

**Figure 3:**
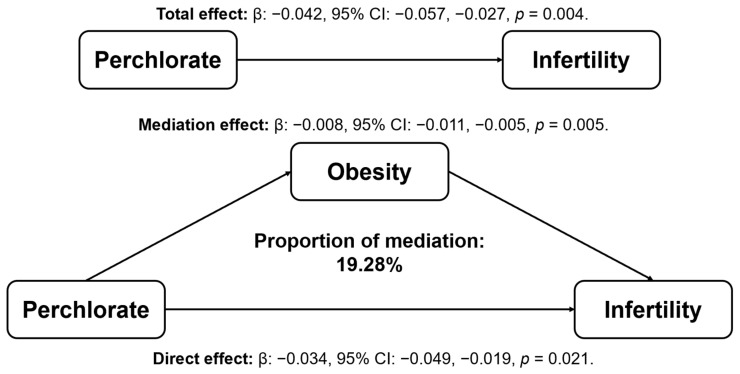
Mediation analysis for associations between perchlorate and infertility. Model was adjusted for age (continuous variable), education level, marital status, economic status, alcohol consumption, smoke status, physical activity, diabetes, hypertension, menstrual cycle regularity, contraceptive pills, and female hormones.

Figure 3 presents the results of mediation analyses. As shown in Figure 3, perchlorate was negatively associated with infertility (total effect: −0.042; 95% CI: −0.057, −0.027; *p* = 0.004). Obesity was identified as a significant mediator in this association, accounting for 19.28% of the mediating effect (mediation effect: −0.008; 95% CI: −0.011, −0.005; *p* = 0.005).

## 4. Discussion

This study investigated the associations of perchlorate, nitrate, and thiocyanate with obesity and infertility in women of reproductive age, as well as the role of obesity as a mediator in the association between perchlorate and infertility. Our results suggest that perchlorate and nitrate were negatively associated with obesity, and perchlorate was also negatively associated with infertility. Mediation analysis further showed that obesity mediated the association between perchlorate and infertility, with a mediation effect of 19.28%.

We found that exposure to perchlorate and nitrate was negatively associated with obesity in women of reproductive age, consistent with findings from previous studies conducted in US adults and children [34,38]. Similarly, other research has reported that perchlorate, nitrate, and thiocyanate was associated with lower waist circumference and BMI in girls [39]. One study found an interesting result that perchlorate was negatively associated with LDL-C elevation [40]. Another study found that perchlorate and thiocyanate were associated with metabolic syndrome, which is contrary to the results of this study [13]. However, the causal relationship between these anions and obesity in women remains unclear, with thyroid function being a possible underlying mechanism. Thyroid hormones regulate many cellular processes involved in resting energy expenditure and basal metabolism [41], and can influence both the total amount and distribution of adipose tissue [42]. Current evidence indicates that higher exposures to perchlorate and nitrate are associated with elevated serum thyroid-stimulating hormone (TSH) levels and lower serum thyroid hormone (TH) levels [23,43]. Additionally, elevated perchlorate levels have been associated with increased sensitivity to central thyroid hormones [44]. Nevertheless, it remains uncertain whether changes in TSH or other thyroid hormones are a cause or consequence of obesity.

We also found that perchlorate was negatively associated with infertility in reproductive-aged women, an effect that may be mediated though thyroid function. Thyroid disease is a common endocrine disorder for reproductive-aged women [45], and affects female reproductive health in several ways, including modulating the HPO axis, regulating the effects of prolactin and leptin on gonadotropin-releasing hormone (GnRH), and altering binding proteins that affect the bioavailability of sex steroids [24,46,47,48,49]. In the 1950s, perchlorate was widely used as a treatment for thyroid dysfunction [50,51,52]. Perchlorate can inhibit the thyroid gland’s ability to take up iodine, which is used to normalize thyroid function in patients with hypothyroidism [53], and has also been used to treat hyperthyroidism [54]. Thus, perchlorate exposure may reduce the risk of infertility by improving thyroid function. The mechanisms by which perchlorate influences infertility are likely to be complex, with obesity potentially playing a critical role in reducing infertility risk. Obesity can adversely affect female reproductive health by disrupting the HPO axis and promoting ovarian androgen production [35,55]. Our study identified a significant mediating effect of obesity in the relationship between perchlorate exposure and infertility, suggesting that obesity management may be a key pathway through which perchlorate exposure helps to reduce the risk of infertility.

Our study has several strengths. First, to our knowledge, this is the first study to investigate the association between exposure to perchlorate, nitrate, and thiocyanate and obesity among reproductive-aged women. Second, it is also the first to investigate the association between these three anions and female infertility. Third, by considering obesity as a mediating variable in the association between these anions and infertility, we offer a novel perspective on infertility treatment strategies. However, this study has some limitations. First, the cross-sectional design of the NHANES data limits our ability to establish causal relationships between the three anions, obesity, and infertility. Second, exposures were assessed using a single urine sample, which may not fully capture the participants’ true exposure levels over time. However, the single-spot urine samples of these anions have shown considerable temporal reliability, making their urine concentrations reliable biomarkers [56,57]. Finally, although we adjusted for several infertility-related risk factors, residual confounding by unmeasured variables may still be present.

## 5. Conclusions

In conclusion, our study found negative associations between perchlorate and nitrate and obesity in women of reproductive age in the US, as well as a negative association between perchlorate and infertility. The inverse association between perchlorate and infertility appears to be mediated by obesity. These findings provide new insights into the health risk assessment of perchlorate, nitrate, and thiocyanate exposure, while offering a novel perspective on strategies for managing female infertility. Further research is needed to confirm these findings and to elucidate the underlying mechanisms.

## Figures and Tables

**Table 1:** Baseline characteristics of the participants.

Characteristics	All Participants	Infertility Status		*p* Value
		Non Infertility	Infertility	
	(N = 813)	(N = 708)	(N = 105)	
Age [years, Mean (SD)]	33.0 (7.4)	32.7 (7.5)	35.1 (6.9)	0.001 ^a^
Race/ethnicity [No. (%)]				0.417 ^c^
White	281 (34.6)	248 (35.0)	33 (31.4)	
Black	170 (20.9)	143 (20.2)	27 (25.7)	
Others	362 (44.5)	317 (44.8)	45 (42.9)	
Education level [No. (%)]				0.313 ^c^
Less than high school	113 (13.9)	100 (14.1)	13 (12.4)	
High school graduate or equivalent	163 (20.0)	147 (20.8)	16 (15.2)	
Above high school	537 (66.1)	461 (65.1)	76 (72.4)	
Marital status [No. (%)]				<0.001 ^c^
Living without partner	466 (57.3)	388 (54.8)	78 (74.3)	
Married/living with partner	347 (42.7)	320 (45.2)	27 (25.7)	
Economic status [No. (%)]				0.700 ^c^
Poverty	285 (35.1)	247 (34.9)	38 (36.2)	
Normal	300 (36.9)	265 (37.4)	35 (33.3)	
Wealthy	228 (28.0)	196 (27.7)	32 (30.5)	
Alcohol consumption [No. (%)]				0.669 ^c^
No	306 (37.6)	264 (37.3)	42 (40.0)	
Yes	507 (62.4)	444 (62.7)	63 (60.0)	
Smoke status [No. (%)]				0.467 ^c^
No	563 (69.2)	494 (69.8)	69 (65.7)	
Yes	250 (30.8)	214 (30.2)	36 (34.3)	
Physical activity [No. (%)]				0.686 ^c^
Sedentary	348 (42.8)	299 (42.2)	49 (46.7)	
Insufficient	119 (14.6)	102 (14.4)	17 (16.2)	
Moderate	93 (11.4)	82 (11.6)	11 (10.5)	
High	253 (31.1)	225 (31.8)	28 (26.7)	
Obesity [No. (%)]				<0.001 ^c^
No	471 (57.9)	429 (60.6)	42 (40.0)	
Yes	342 (42.1)	279 (39.4)	63 (60.0)	
Diabetes [No. (%)]				0.045 ^c^
No	783 (96.3)	686 (96.9)	97 (92.4)	
Yes	30 (3.7)	22 (3.1)	8 (7.6)	
Hypertension [No. (%)]				0.002 ^c^
No	697 (85.7)	618 (87.3)	79 (75.2)	
Yes	116 (14.3)	90 (12.7)	26 (24.8)	
Menstrual cycle regularity [No. (%)]				0.197 ^c^
No	743 (91.4)	651 (91.9)	92 (87.6)	
Yes	70 (8.6)	57 (8.1)	13 (12.4)	
Contraceptive pills [No. (%)]				0.330 ^c^
No	238 (29.3)	212 (29.9)	26 (24.8)	
Yes	575 (70.7)	496 (70.1)	79 (75.2)	
Female hormones [No. (%)]				0.378 ^c^
No	786 (96.7)	686 (96.9)	100 (95.2)	
Yes	27 (3.3)	22 (3.1)	5 (4.8)	
Urine Perchlorate [ug/g Cr, median (IQR)]	2.45 (1.54, 4.09)	2.52 (1.56, 4.32)	2.07 (1.30, 3.52)	0.008 ^b^
Urine Nitrate [mg/g Cr, median (IQR)]	45.77 (34.43, 67.56)	46.08 (34.24, 67.36)	45.51 (35.90, 72.44)	0.918 ^b^
Urine Thiocyanate [mg/g Cr, median (IQR)]	1.18 (0.63, 2.42)	1.19 (0.62, 2.43)	1.10 (0.65, 2.40)	0.988 ^b^

Abbreviation: Cr: creatinine. ^a^: Student’s *t*-test was used for normally distributed continuous variables. ^b^: Mann–Whitney U test was used for the skewed variables. ^c^: Chi-square test was used for categorical variables.

**Table 2:** Associations between perchlorate, nitrate, and thiocyanate exposures and obesity.

Exposures		Ln-Transformed	Q1 OR (95% CI)	Q2 OR (95% CI)	Q3 OR (95% CI)	Q4 OR (95% CI)	*p* Trend
		OR (95% CI), *p* Value	*p* Value	*p* Value	*p* Value	*p* Value	
Perchlorate	Model 1	0.70 (0.53–0.93)	1 [Reference]	0.87 (0.50–1.51)	0.66 (0.38–1.15)	0.50 (0.27–0.91)	0.016
		0.017	Ref.	0.620	0.152	0.028	
	Model 2	0.73 (0.55–0.96)	1 [Reference]	0.88 (0.51–1.54)	0.72 (0.41–1.27)	0.53 (0.30–0.96)	0.032
		0.031	Ref.	0.660	0.266	0.045	
	Model 3	0.73 (0.55–0.96)	1 [Reference]	0.86 (0.50–1.48)	0.74 (0.42–1.29)	0.54 (0.30–0.96)	0.037
		0.032	Ref.	0.598	0.295	0.045	
Nitrate	Model 1	0.60 (0.40–0.90)	1 [Reference]	0.83 (0.48–1.47)	0.85 (0.48–1.52)	0.45 (0.24–0.84)	0.016
		0.018	Ref.	0.533	0.597	0.018	
	Model 2	0.61 (0.40–0.92)	1 [Reference]	0.85 (0.51–1.40)	0.93 (0.55–1.56)	0.46 (0.24–0.86)	0.023
		0.024	Ref.	0.525	0.775	0.020	
	Model 3	0.59 (0.40–0.87)	1 [Reference]	0.88 (0.55–1.42)	1.00 (0.58–1.71)	0.45 (0.24–0.83)	0.029
		0.013	Ref.	0.610	0.999	0.016	
Thiocyanate	Model 1	1.01 (0.86–1.20)	1 [Reference]	0.84 (0.55–1.30)	0.74 (0.42–1.29)	1.10 (0.67–1.80)	0.799
		0.880	Ref.	0.445	0.296	0.716	
	Model 2	0.91 (0.77–1.07)	1 [Reference]	0.87 (0.57–1.34)	0.82 (0.47–1.42)	0.88 (0.55–1.39)	0.526
		0.268	Ref.	0.539	0.478	0.582	
	Model 3	0.92 (0.77–1.11)	1 [Reference]	0.90 (0.59–1.37)	0.82 (0.46–1.46)	0.92 (0.56–1.51)	0.646
		0.398	Ref.	0.630	0.515	0.741	

Abbreviation: OR: odds ratio, CI: confidence intervals; Q: quartile. Model 1 was adjusted for age and race. Model 2 further controlled for education level, marital status, economic status, alcohol consumption, smoke status, and physical activity. Model 3 further controlled for diabetes, hypertension, menstrual cycle regularity, contraceptive pills, and female hormones.

**Table 3:** Associations between perchlorate, nitrate, and thiocyanate exposures and infertility.

Exposure		Ln-Transformed	Q1 OR (95% CI)	Q2 OR (95% CI)	Q3 OR (95% CI)	Q4 OR (95% CI)	*p* Trend
		OR (95% CI), *p* Value	*p* Value	*p* Value	*p* Value	*p* Value	
Perchlorate	Model 1	0.66 (0.50–0.88)	1 [Reference]	1.23 (0.61–2.47)	0.86 (0.43–1.72)	0.46 (0.23–0.94)	0.015
		0.007	Ref.	0.571	0.678	0.040	
	Model 2	0.65 (0.48–0.86)	1 [Reference]	1.34 (0.67–2.69)	0.85 (0.42–1.73)	0.46 (0.22–0.94)	0.015
		0.006	Ref.	0.420	0.655	0.042	
	Model 3	0.68 (0.51–0.91)	1 [Reference]	1.32 (0.64–2.72)	0.85 (0.42–1.72)	0.45 (0.23–0.90)	0.013
		0.016	Ref.	0.453	0.653	0.034	
Nitrate	Model 1	0.97 (0.67–1.39)	1 [Reference]	1.31 (0.63–2.69)	0.80 (0.40–1.59)	1.03 (0.48–2.20)	0.763
		0.858	Ref.	0.476	0.526	0.944	
	Model 2	0.91 (0.63–1.32)	1 [Reference]	1.21 (0.59–2.47)	0.72 (0.36–1.43)	0.86 (0.40–1.85)	0.455
		0.627	Ref.	0.606	0.350	0.699	
	Model 3	1.02 (0.68–1.53)	1 [Reference]	1.26 (0.62–2.56)	0.78 (0.40–1.53)	1.02 (0.43–2.41)	0.796
		0.930	Ref.	0.534	0.471	0.973	
Thiocyanate	Model 1	0.95 (0.77–1.17)	1 [Reference]	1.48 (0.68–3.21)	0.98 (0.49–1.97)	0.91 (0.46–1.79)	0.410
		0.610	Ref.	0.330	0.954	0.784	
	Model 2	0.85 (0.67–1.07)	1 [Reference]	1.32 (0.62–2.82)	0.84 (0.42–1.68)	0.62 (0.31–1.22)	0.089
		0.177	Ref.	0.484	0.624	0.174	
	Model 3	0.88 (0.67–1.16)	1 [Reference]	1.54 (0.69–3.46)	0.89 (0.42–1.89)	0.70 (0.33–1.47)	0.188
		0.372	Ref.	0.301	0.765	0.353	

Abbreviation: OR: odds ratio, CI: confidence intervals; Q: quartile. Model 1 was adjusted for age and race. Model 2 further controlled for education level, marital status, economic status, alcohol consumption, smoke status, and physical activity. Model 3 further controlled for diabetes, hypertension, menstrual cycle regularity, contraceptive pills, and female hormones.

## Data Availability

The original datasets supporting the analysis are publicly available on the NHANES website (https://www.cdc.gov/nchs/nhanes (accessed on 1 September 2024)). The analysis datasets and programming code are accessible from the corresponding author and will be provided if reasonably requested.

## Supplementary Materials

The following supporting information can be downloaded at: https://www.mdpi.com/article/10.3390/toxics13010015/s1, Figure S1: Spearman correlations among perchlorate, nitrate, and thiocyanate; Table S1: Associations between perchlorate, nitrate, thiocyanate exposures and infertility (sensitivity analysis i–v).
