# Supplementary material for: Associations of Urinary Perchlorate, Nitrate, and Thiocyanate with Female Infertility and Mediation of Obesity: Insights from NHANES 2013–2018
Source: Toxics. 2024 Dec 26;13(1):15. doi: 10.3390/toxics13010015 (PMC11769535; doi:10.3390/toxics13010015)
Supplement: Supplementary file 1 [file toxics-13-00015-s001.zip › toxics-3352082-supplementary.pdf]

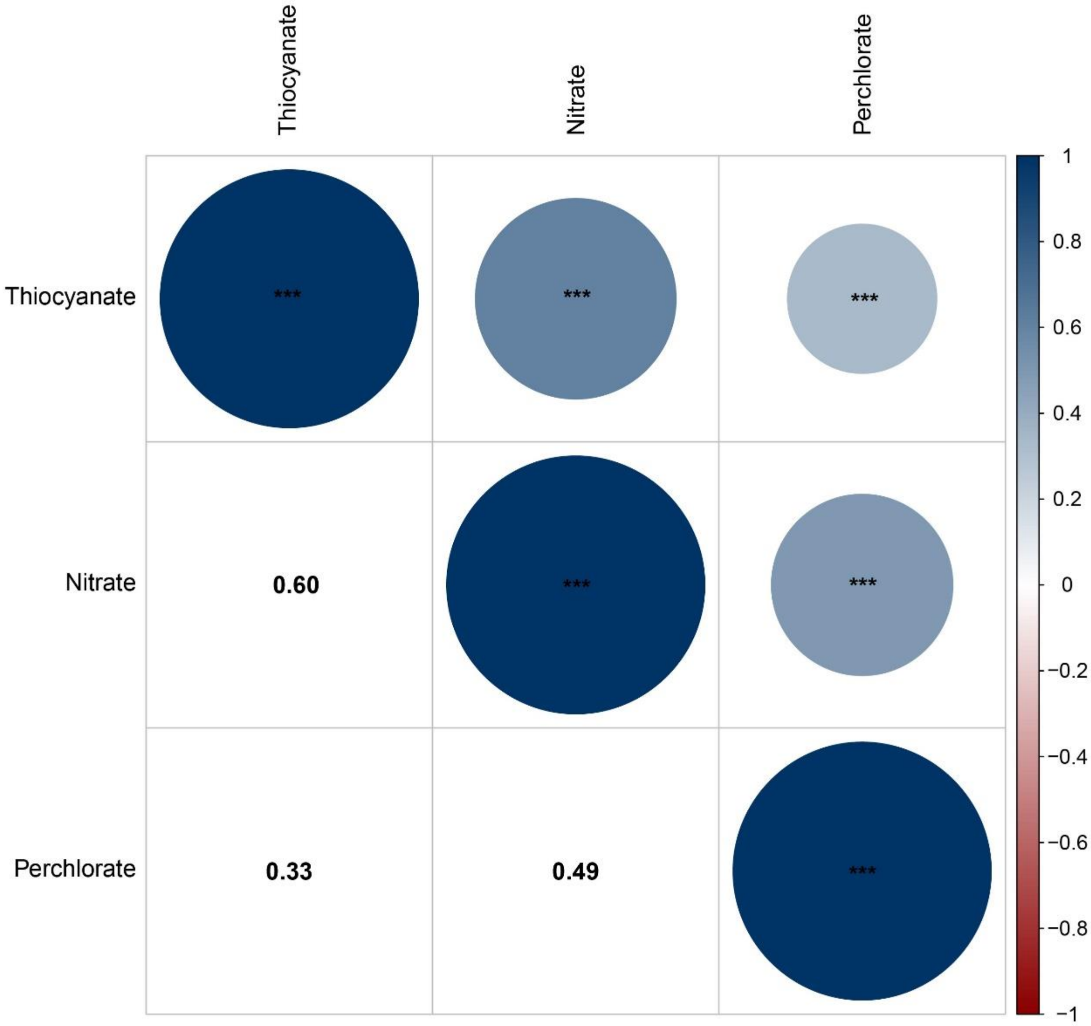

Supplemental Figure S1. Spearman correlations among perchlorate, nitrate, and thiocyanate.
*** means P < 0.001

Supplemental Table S1. Associations between perchlorate, nitrate, thiocyanate exposures and infertility (sensitivity analysis i-v).

Sensitivity group	Perchlorate		Nitrate		Thiocyanate	
	Odds ratio (95%CI)	P Value	Odds ratio (95%CI)	P Value	Odds ratio (95%CI)	P Value
Sensitivity analysis i	0.66 (0.49-0.88)	0.005	1.14 (0.82-1.61)	0.433	1.00 (0.79-1.26)	0.992
Sensitivity analysis ii	0.62 (0.45-0.85)	0.007	1.16 (0.77-1.77)	0.484	0.87 (0.67-1.14)	0.334
Sensitivity analysis iii	0.72 (0.53-0.98)	0.048	1.13 (0.79-1.62)	0.518	0.95 (0.72-1.25)	0.725
Sensitivity analysis iv	0.70 (0.54-0.92)	0.017	0.91 (0.64-1.28)	0.586	0.87 (0.68-1.11)	0.271
Sensitivity analysis v	0.64 (0.45-0.89)	0.014	0.85 (0.48-1.50)	0.573	0.76 (0.57-1.02)	0.083

Sensitivity analysis i conducted multivariable logistic regression without applying NHANES weights. Sensitivity analysis ii further adjusted for urinary perchlorate, nitrate, and thiocyanate in model 3. Sensitivity analysis iii included creatinine as a covariate in the models. Sensitivity analysis iv used imputed data. Sensitivity analysis v excluded extreme values from the top and bottom 1%.

The model was adjusted for age (continuous variable), education level, marry status, economic status, alcohol consumption, smoke status, physical activity, diabetes, hypertension, menstrual cycle regularity, contraceptive pills, and female hormones.
